# Renal Cell Carcinoma-Infiltrating CD3^low^ Vγ9Vδ1 T Cells Represent Potentially Novel Anti-Tumor Immune Players

**DOI:** 10.3390/cimb43010019

**Published:** 2021-05-27

**Authors:** Hye Won Lee, Chanho Park, Je-Gun Joung, Minyong Kang, Yun Shin Chung, Won Joon Oh, Seon-Yong Yeom, Woong-Yang Park, Tae Jin Kim, Seong Il Seo

**Affiliations:** 1 Department of Urology, Center for Urologic Cancer, National Cancer Center, Goyang 10408, Korea; nsproper@naver.com; 2 Department of Immunology, Sungkyunkwan University School of Medicine, Suwon 16419, Korea; darkstar0113@hanmail.net (C.P.); chungys81@gmail.com (Y.S.C.); joon516@skku.edu (W.J.O.); 3 Department of Biomedical Science, College of Life Science, CHA University, Seongnam 13488, Korea; jegunjoung@gmail.com; 4 Samsung Medical Center, Department of Urology, Sungkyunkwan University School of Medicine, Seoul 06351, Korea; m79.kang@samsung.com (M.K.); skyone0802@hanmail.net (S.-Y.Y.); 5 Department of Health Science and Technology, Samsung Advanced Institute for Health Science and Technology, Sungkyunkwan University, Seoul 06351, Korea; woongyang.park@samsung.com

**Keywords:** renal cell carcinoma, tumor microenvironment, tumor-infiltrating lymphocytes, gamma-delta T cells, anti-tumor immunity, adoptive immunotherapy

## Abstract

Due to the highly immunogenic nature of renal cell carcinoma (RCC), the tumor microenvironment (TME) is enriched with various innate and adaptive immune subsets. In particular, gamma-delta (γδ) T cells can act as potent attractive mediators of adoptive cell transfer immunotherapy because of their unique properties such as non-reliance on major histocompatibility complex expression, their ability to infiltrate human tumors and recognize tumor antigens, relative insensitivity to immune checkpoint molecules, and broad tumor cytotoxicity. Therefore, it is now critical to better characterize human γδ T-cell subsets and their mechanisms in RCCs, especially the stage of differentiation. In this study, we aimed to identify γδ T cells that might have adaptive responses against RCC progression. We characterized γδ T cells in peripheral blood and tumor-infiltrating lymphocytes (TILs) in freshly resected tumor specimens from 20 RCC patients. Furthermore, we performed a gene set enrichment analysis on RNA-sequencing data from The Cancer Genome Atlas (TCGA) derived from normal kidneys and RCC tumors to ascertain the association between γδ T-cell infiltration and anti-cancer immune activity. Notably, RCC-infiltrating CD3^low^ Vγ9Vδ1 T cells with a terminally differentiated effector memory phenotype with up-regulated activation/exhaustion molecules were newly detected as predominant TILs, and the cytotoxic activity of these cells against RCC was confirmed in vitro. In an additional analysis of the TCGA RCC dataset, γδ T-cell enrichment scores correlated strongly with those for CTLs, Th1 cells, “exhausted” T cells, and M1 macrophages, suggesting active involvement of γδ T cells in anti-tumor rather than pro-tumor activity, and Vδ1 cells were more abundant than Vδ2 or Vδ3 cells in RCC tumor samples. Thus, we posit that Vγ9Vδ1 T cells may represent an excellent candidate for adoptive immunotherapy in RCC patients with a high risk of relapse after surgery.

## 1. Introduction

Postoperative relapse develops in a significant proportion of patients with organ-confined renal cell carcinoma (RCC) after curative surgery, and they have a universally unfavorable prognosis due to treatment failure [1]. Surgical rection of primary tumors removes anti-tumor immune effectors and induces wound healing to initiate new metastases [2]. On the other hand, in advanced RCC with limited metastasis, a cytoreductive nephrectomy maintains a role in reducing the tumor burden and symptoms [1]. New immunotherapeutic strategies in the perioperative setting before the reestablishment of a concentrated immunosuppressive tumor microenvironment (TME) are warranted for successful adjuvant or palliative therapeutics that may thus confer a durable survival benefit in patients with RCC.

Additional progress will depend on understanding the immunobiology of RCC, which is complex and different from that of other immune-sensitive tumor types [3,4,5]. RCC exhibits the highest number of insertion-deletion mutations among all cancer types [5]. Consistent with such high immunogenicity, the TME of most RCCs, particularly that of the clear cell subtype, demonstrates abundant innate and adaptive immune cell infiltrates [3,4,5,6]. γδ T cells are a group of heterogeneous T cells composed of various subgroups based on their T-cell receptor (TCR) composition and cellular function, accounting for 0.5–5% of all T cells [7,8,9]. As γδ T cells interact with different types of innate and adaptive immune cells in the TME and modulate the anti-tumor response, pleiotropic effects of the mixture of both anti- and pro-tumor γδ T cells used in the adoptive cell transfer (ACT) may be induced by the immunosuppressive TME [7,8,9,10]. μδ T cells recognize and respond to a broad range of tissue-specific tumor-associated antigens, including phosphorylated metabolites arising from an altered tumor metabolism, and stress-associated antigens in cancer cells produced via γδ TCRs or natural killer (NK) cell receptors [7,8,9]. These unique properties make γδ T cells attractive mediators of cancer immunotherapy as ACT.

Unfortunately, recent clinical trials have shown that although applications of γδ T cells to advanced solid tumors, including RCCs, yield promising clinical benefits and safety, obvious limitations remain, with an average response ratio of only 21% and a low proportion of complete remissions [7,8,11]. Therefore, the present study focused on a better characterization of tumor-infiltrating γδ T cell subsets and their mechanisms in RCCs, especially in the stage of differentiation, activation status, and clinically relevant anti-tumor functions to irreversibly convert them into anti-tumor effectors to develop an efficient combination of immunotherapeutic strategies.

## 2. Materials and Methods

### 2.1. Patients and Clinical Samples

We evaluated treatment-naïve patients with histologically confirmed RCC after partial or radical nephrectomy at Samsung Medical Center (SMC, Seoul, Korea) from 2018 to 2019. All analyses were approved by the appropriate Institutional Review Board (IRB) at SMC (IRB No: 2018-04-037). Written informed consent was obtained from all patients. Heparinized peripheral blood and sections of fresh tumor tissue (from non-necrotic, non-hemorrhagic central regions) were collected during surgeries. 

### 2.2. Isolation of Tumor-Infiltrating Lymphocytes (TILs) and Matched Peripheral Blood Lymphocytes (PBLs) from Patients with RCC

Explanted fresh tumor tissue was rinsed with phosphate-buffered saline to remove traces of blood and necrosis. The material, diced using scalpels, was filtered through a fine (70 μm) mesh of cell strainer (SPL Life Sciences Co., Pocheon-si, Gyeonggi-do, Korea), and red blood cell (RBC) lysis buffer (BioLegend, San Diego, CA, USA) was used to lyse RBCs with minimal effect on white blood cells after centrifugation at 2000 rpm for 4 min. RCC TILs were finally isolated by repeated centrifugation (2000 rpm; 4 min), and the cell suspension was immediately analyzed after assessing cell viability using trypan blue exclusion. Subsequently, PBLs were enriched from heparinized peripheral blood collected from matched patients using the Ficoll-Paque^TM^ (GE Healthcare, Chicago, IL, USA) gradient method as described previously [12].

### 2.3. Immunophenotyping Using Flow Cytometry of TILs and PBLs from Patients with RCC

For phenotyping, freshly isolated TILs and PBLs (10^5^–10^7^) were labelled without stimulation or expansion using a panel of monoclonal antibodies specific for differentiation, activation, exhaustion, and cytotoxic molecules: anti-human antibodies against CD3 (HIT3a), γδTCR (B1), CD28 (CD28.2), Fas Cell Surface Death Receptor (FAS) (DX2), Vγ9 (B3) Vδ2 (B6) Programmed cell death protein 1 (PD-1) (EH12.2H7), Inducible T-cell co-stimulator (ICOS) (C398.4A), CD69 (FN50), CD27 (M-T271), CD45RA (HI100), B- and T-lymphocyte attenuator (BTLA) (MIH26), Lymphocyte Activating 3 (LAG3) (11C3C65), T-cell immunoglobulin and mucin domain-3 (TIM-3) (F38-2E2), T-cell immunoreceptor with Ig and ITIM domains (TIGIT) (A15153G), NK group 2 member D (NKG2D) (1D11), Perforin (B-D48), Granzyme A (CB9), Granzyme B (GB11), and Lysosomal-associated membrane protein 1 (LAMP-1) (H4A3) (all purchased from BioLegend, San Diego, CA, USA) and Vδ1 (TS8.2) (purchased from Thermo Fisher Scientific, Waltham, MA, USA). Cells were stained with 0.25 μg/mL human BD Fc Block (BD Biosciences, San Jose, CA, USA) to block Fcγ receptors, and concentration- and isotype-matched control antibodies were used to detect the nonspecific binding of antibodies. After a 30-min incubation on ice followed by two PBS washes, cells were analyzed using the BD FACS CantoII^TM^ flow cytometer (BD Biosciences, San Diego, CA, USA) and FlowJo software (BD Biosciences, San Diego, CA, USA) was used for data acquisition and analysis.

### 2.4. Cytotoxic Assay with Human RCC Cell Lines and γδ T Cells

For cytotoxicity assessments, human RCC cell lines Caki-1 (metastatic, clear cell subtype, *Von Hippel–Lindau* (*VHL*) gene wild-type, well-differentiated) and ACHN (metastatic, papillary subtype, *VHL* gene wild-type, poorly differentiated) (4 × 10^4^ cells/well) were incubated overnight in 48-well plates. Isolated RCC infiltrating Vγ9Vδ1 T cells or peripheral Vγ9Vδ2 T cells (5 × 10^3^~1 × 10^4^ cells/well) were added to target the tumor cells per well with and without activation. RCC-infiltrating Vγ9Vδ1 T cells were stimulated with anti-human Vδ1 Ab. For the cytotoxicity assay with Vγ9Vδ2 T cells, RCC cell lines were treated with hydroxymethylbutenyl-4-diphosphate (HMB-PP) for 20 h prior to performing cytotoxicity assays. After incubation for 24 h at 37 °C, tumor cell death was measured by eBioscience^TM^ Fixable Viability Dye eFluor^TM^ 780 (Thermo Fisher Scientific, Waltham, MA USA) staining as per manufacturer’s instructions and analyzed by flow cytometry (BD FACS CantoII^TM^ flow cytometer (BD Biosciences, San Jose, CA, USA) and FlowJo software software (BD Biosciences, San Diego, CA, USA).

### 2.5. Statistical Analysis

Statistical analysis was performed using GraphPad Prism software (Prism Software, Lake Forest, CA, USA). Results are expressed as mean ± standard deviation (SD). The statistical significance of differences between groups was determined using a *t*-test or one-way analysis of variance (ANOVA). Statistical significance is indicated when *p* < 0.05; * *p* < 0.05, ** *p* < 0.01, *** *p* < 0.001, and **** p < 0.0001 in the Figures.

## 3. Results

### 3.1. Elucidation of the Immunological Characteristics of a Novel γδ T Subset in RCC TME

**Figure 1:**
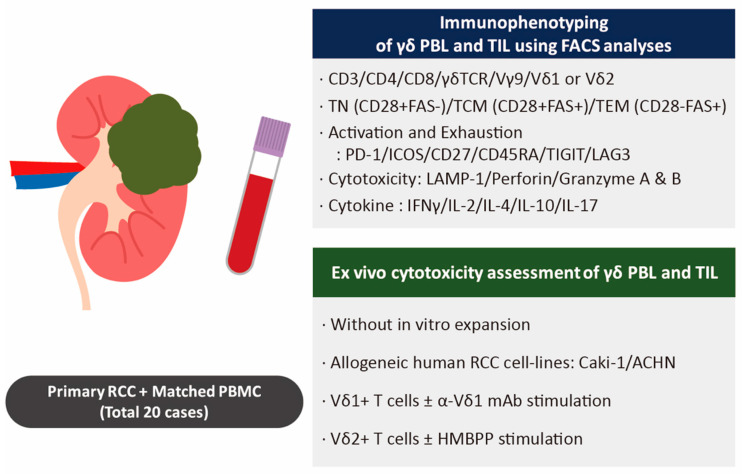
Analysis of γδ T cells within renal cell carcinoma (RCC) and patients’ blood. Experimental schemes: Tumor-infiltrating and blood lymphocytes were analyzed as indicated (TN, T naïve; TCM, central memory T cell; TEM, effector memory T cell). The cytotoxic capacity of each of the sorted γδ T cell populations was assessed for their cytotoxic capacity against Caki-1 and ACHN RCC cell lines.

**Figure 2:**
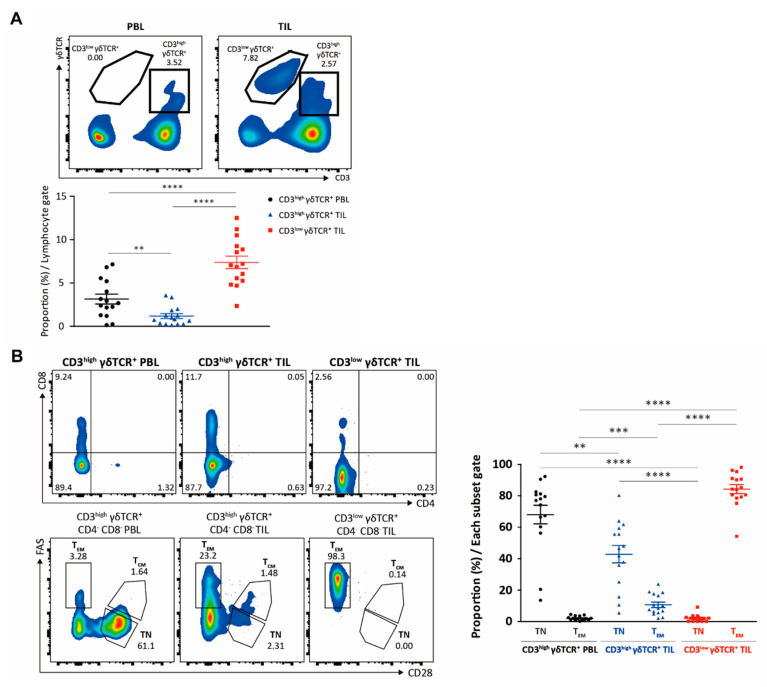
Analysis of peripheral blood (PB) and tumor-infiltrating lymphocytes (TILs). (**A**) PB and TILs were analyzed for γδ TCR and CD3. (**B**) CD3^high^ γδ T cells from peripheral blood lymphocytes (PBLs) and TILs and CD3^low^ γδ T cells from TILs were analyzed for CD4 and CD8, as well as for FAS and CD28. ** *p* < 0.01, *** *p* < 0.001, and **** p < 0.0001.

We characterized γδ T cell populations in peripheral blood and freshly resected tumor specimens from 20 patients with RCC (Figure 1 and Table 1). We observed fewer circulating than tumor-infiltrating γδ T cells, and based on CD3 expression, two intra-tumoral γδ T cell subpopulations were distinguishable (Figure 2a). While almost all circulating γδ T cells were CD3^high^, intra-tumoral γδ T cells could be classified as CD3^low^ (the major population) or CD3^high^ (the minor population), both largely CD4^-^CD8^-^ (Figure 2b). Over 80% of intra-tumoral CD3^low^ γδ T cells were of the FAS^+^CD28^-^ effector memory type, suggesting a state of chronic activation, whereas the majority of blood CD3^high^ γδ T cells were naïve (Figure 2b). Less than 20% of intra-tumoral CD3^high^ γδ T cells were of this memory type.

**Figure 3:**
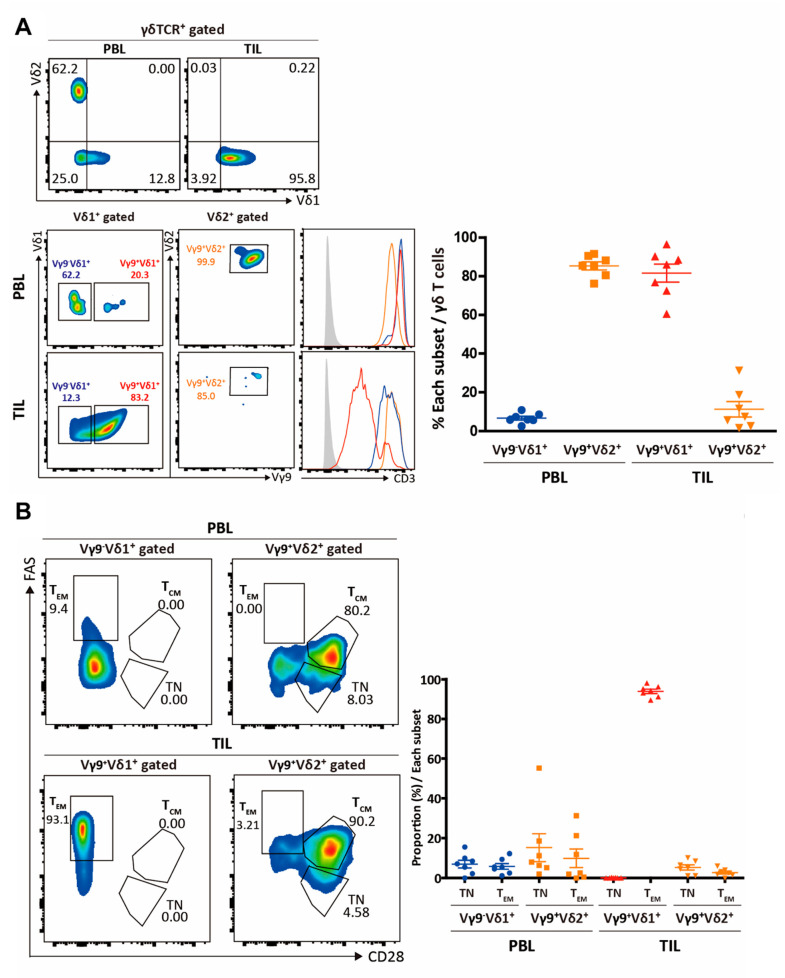
Identification of intra-tumoral CD3^low^ γδ T cells. (**A**) Peripheral blood lymphocytes (PBLs) and TIL γδ T cells were assessed for the expression of Vδ1 and Vδ2 chains, while gated Vδ1 and Vδ2 γδ T cells were analyzed for the expression of Vγ9 chain. The level of the CD3 expression is shown for Vγ9-δ1+, Vγ9+-δ1+, and Vγ9+-δ2+ T cells. (**B**) Gated Vγ9+-δ1+ and Vγ9+-δ2+ T cells in PBLs or TILs were analyzed for FAS and CD28 to assess their differentiation statuses.

Notably, intra-tumoral CD3^low^ γδ T cells were of the Vγ9δ1 subtype, distinct from circulating CD3^high^ Vγ9δ2 T cells (Figure 3A). As expected, ~80% of the circulating γδ T cells were of the Vγ9δ2 subtype, while only ~10% of intra-tumoral γδ T cells were of the Vγ9δ2 subtype. Intratumorally, the expression of CD3 was low only among Vγ9δ1 T cells (not among Vδ1 cells lacking the Vγ9 chain or among Vγ9δ2 T cells), indicating chronic TCR-mediated activation within the RCC TME. Consistent with this observation, intra-tumoral Vγ9δ1 T cells were FAS^+^CD28^-^ effector memory cells expressing immune checkpoint receptors including PD-1, ICOS, CD45RA, LAG3, TIGIT, and TIM3 [13,14], consistent with a terminally differentiated effector memory phenotype (CD28^-^ FAS^+^ CD45RA^+^ CD27^−^) (Figure 3B). They also expressed CD45RA, normally a marker of naivety, but here a feature of effector memory T cells re-expressing CD45RA (T_EMRA_) that occur in both the CD4^+^ and CD8^+^ compartments of αβ T cells. 

**Figure 4:**
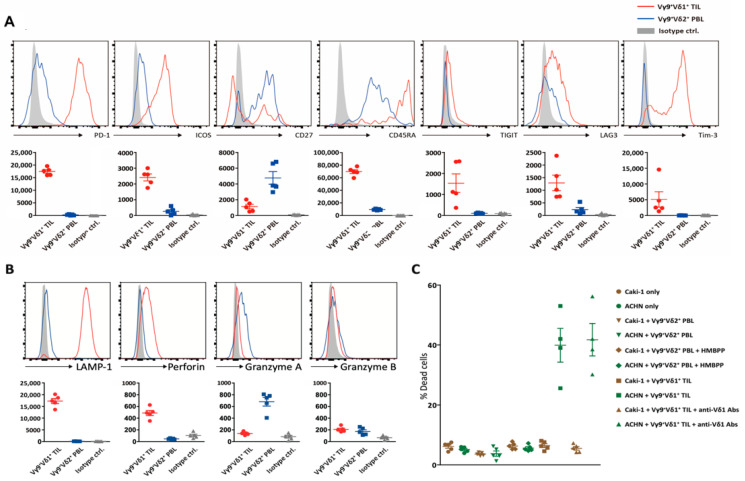
Immune modulation and cytotoxic activity. (**A**) The proportions of dead cells were shown for the cytotoxicity assays using two RCC cell lines and sorted γδ T cell populations as mean ± SD. (**B**) Vγ9+-δ1+ TILs and Vγ9+-δ2+ peripheral blood lymphocytes (PBLs) were compared for the expression of co-stimulatory molecules (ICOS and CD27), immune checkpoint receptors (PD-1, TIGIT, LAG3, and TIM-3) and CD45RA. Shaded histograms show the staining of the negative controls. Values from all patients are shown as dot graphs with mean ± SD and statistical significances. (**C**) Cytotoxic activity of intra-tumoral Vγ9δ1 T cells.

In addition, up-regulation of both activation/exhaustion molecules suggested the potent anti-tumor cytotoxic activity of these Vγ9δ1 T cells (Figure 4A,B) [7,8,9,10,11,15,16]. In general, human memory T cell differentiation follows a linear progression along a continuum of major clusters, where less differentiated cells give rise to more differentiated progeny in response to antigenic stimulation [13]. Immune effectors usually designate T_EMRA_ capable of immediate cytokine production and cytotoxicity [13]. Supporting a previous report, in this study, intra-tumoral Vγ9δ2 T cells secreted a different set of cytotoxic mediators (granzymes A and B), indicating different stages of differentiation for Vγ9δ1 and Vγ9δ2 cells within the RCC TME (Figure 4B). The cytotoxic activity of intra-tumoral Vγ9δ1, but not Vγ9δ2, T cells against RCC was verified in vitro, with the former cell type able to lyse ACHN but not Caki-1 cells (Figure 4C).

**Figure 5:**
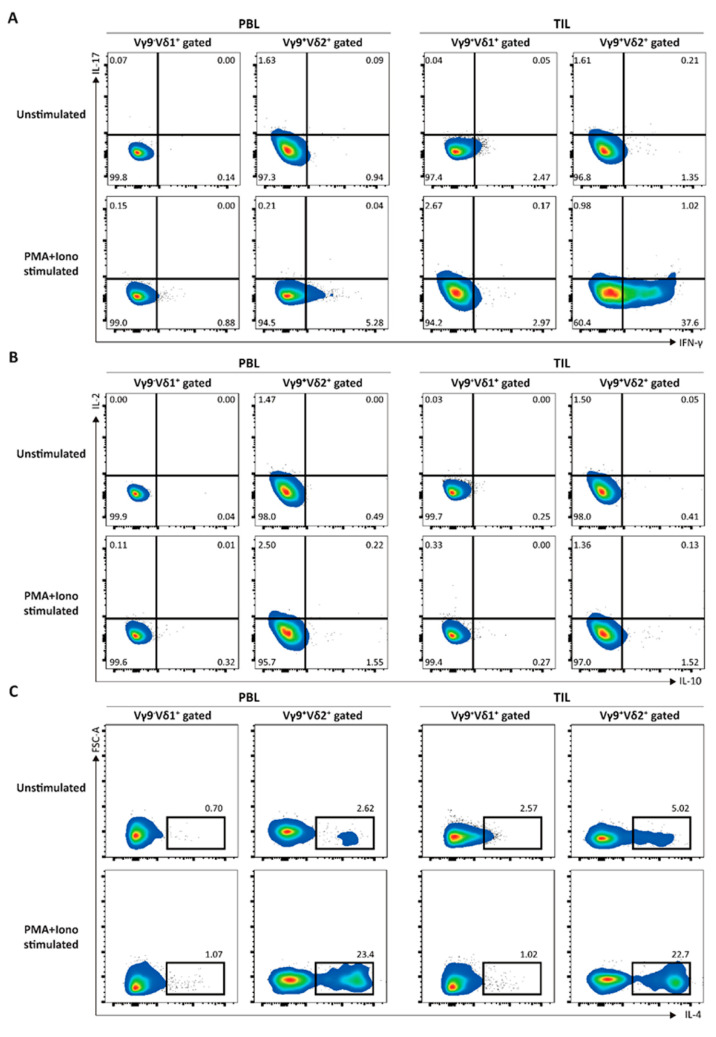
Cytokine secretion by Vγ9+-δ1+ or Vγ9+-δ2+ cells. Lymphocytes from peripheral blood (PBL) or tumor (TIL) stimulated with or without phorbol myristate acetate (PMA) and ionomycin for 4 hours were stained with Abs against indicated cytokines; (**A**) IL-17 and IFN-γ, (**B**) IL-2 and IL-10, and (**C**) IL-4. The cytokine expression was shown for the gated Vγ9+-δ1+ or Vγ9+-δ2+ cells.

The bias of intra-tumoral Vγ9δ1 T cells toward cytotoxicity rather than cytokine production, as demonstrated by an inability to secrete cytokines in response to either phorbol myristate acetate or ionomycin (Figure 5), suggests a terminally differentiated and “exhausted” phenotype that minimizes damage to healthy host tissue while retaining anti-tumor activity. Furthermore, the inability to secrete interferon-γ or other cytokines may decrease the pro-tumoral aspects of the TME [17].

### 3.2. External Validation of the Anti-Cancer Properties of Newly Detected RCC-Infiltrating Vγ9δ1 T Cells via Bioinformatics Analysis

**Figure 6:**
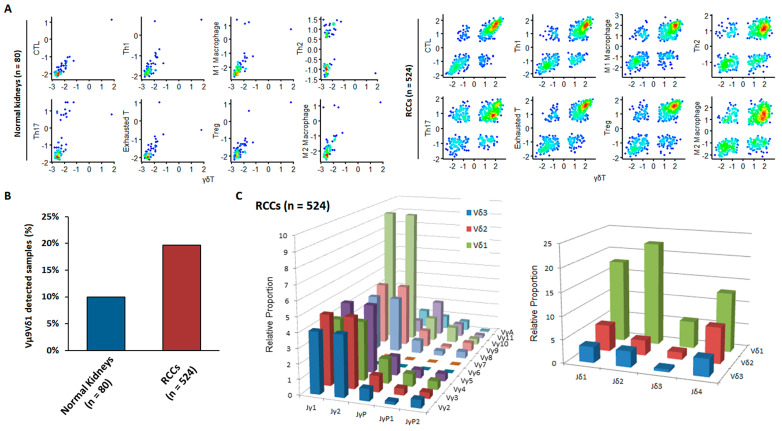
Analysis of immune gene signatures and TCR repertoire for 524 Kidney Renal Clear Cell Carcinoma patients from The Cancer Genome Atlas database. (**A**) Scatter plots of Gene Set Enrichment Analysis enrichment scores between γδ T cell and other immune gene signatures. (**B**) Percentage of samples in which Vγ9Vδ1 clone was detected using RNA-seq data. (**C**) The relative proportion of clones with the combination of Vγ and Jγ segments (left) and combination of Vδ and Jδ segments combination (right) in tumor samples.

It is unclear whether a progenitor population replenishes terminally differentiated Vγ9δ1 T cells similar to the progenitor-mediated replacement of exhausted anti-tumor CD8^+^ T cells. To confirm the association between γδ T cell infiltration and cytotoxic T lymphocyte (CTLs) activity, we performed a GSEA on RNA-seq data from TCGA derived from normal kidneys (*n* = 72) and RCC tumors (*n* = 524) (Figure 6A). Immune gene signature enrichment scores for γδ T cells were plotted against those for other immune cell types (immune signature gene sets are listed in Table 2). γδ T cell enrichment scores were more strongly correlated with those for CTLs, Th1 cells, “exhausted” T cells, and M1 macrophages than with those for Th2 cells, Th17 cells, regulatory T cells, or M2 macrophages, supporting our novel findings on the active involvement of γδ T cells in anti-tumoral rather than pro-tumoral activity.

Importantly, within the TCGA dataset, both TCR Vγ9- and Vδ1-chain sequences were detectable in ~20% of RCC but in only ~10% of normal kidney samples (Figure 6B). Consistent with our flow cytometry data, Vδ1 cells were more abundant than Vδ2 or Vδ3 cells within RCC tumor samples (Figure 6C). In humans, seven functional Vγ gene segments, Vγ2, Vγ3, Vγ4, Vγ5, Vγ8, Vγ9, and Vγ11, are used for the rearrangement of the γ chain [7,8,9,10,15]. Comparable expression levels of Vγ2, Vγ3, Vγ4, Vγ8, and Vγ9 chains within tumors suggest that several Vδ1 cells with different γ chains are present simultaneously within the TME (Vγ5 and Vγ10 sequences are pseudogenes and were excluded from the analysis). One discrepancy was at a higher level of Vγ9 chain expression in our data than in the TCGA dataset. A paucity of blood-derived Vγ9Vδ2 T cells among tumor-infiltrating γδ T cells was inferred from the lower levels of Vγ9JγP than those of Vγ9Jγ1 or Vγ9Jγ2 chain expression (circulating canonical Vγ9Vδ2 T cells express the Vγ9JγP combination) [13].

## 4. Discussion

RCC-infiltrating γδ T cells are diverse, comprising either prominent Vδ1, Vγ9Vδ2, or mixed Vδ subfamilies [7,8,9,15] but at very low frequencies. The Vδ1 and Vγ9Vδ2 T cell subsets develop at different stages during RCC progression, consistent with a distinct underlying TCR repertoire and immunobiology associated with tissue localization and activation modes associated with the induction, polarization, and/or regulation of immune responses [7,8,9,15]. Interestingly, as human Vγ9Vδ2 T cells are among the best understood predominant peripheral blood subsets and can readily be expanded and manipulated ex vivo using PBLs, most of these clinical trials were performed with activated and expanded Vγ9Vδ2 T cells [7,8,11]. Compared to Vγ9δ2 T cells, Vδ1 T cells are a minor subset with distinct innate recognition and regulatory properties that possess enhanced powerful tumoricidal activity, are less susceptible to activation-induced cell death, and live longer [7,8,10,11,15,18], which are in favor of a durable effector function. However, our current understanding of human Vγ9δ2 T cells is primarily based on peripheral blood subsets, while the immunobiology of tumor tissue-associated Vδ1 cells have been mostly uncharacterized. We are just beginning to explore the potential therapeutic role of Vδ1 T cells.

In this study, we conducted the first comprehensive immunophenotypic analysis of γδ T cell subsets isolated from RCC tumor tissue and matched PBL and assessed their anti-tumor cytotoxicity to allogeneic human RCC tumor cell lines. TILs and PBLs isolated from patients with treatment-naïve RCCs were immediately assessed without any manipulation because activating and expanding γδ T cells ex vivo may induce changes in the phenotypic characterization and cellular function of these naturally occurring cells capable of recognizing RCC in vivo. The TCR in γδ T cells consists of TCRγδ and CD3 subunits (CD3γ, δ, ε, and ζ), and γδ T cells have certain unique features in the TCR/CD3 complex and its downstream signaling pathways that dictate their maturation and effector function [7,8,11,19]. Although infiltrations of CD3^high^ Vδ1 T cells and CD3^high^ Vγ9δ2 T cells have been reported in RCC [7,8,9,10,11,19], CD3 membrane density could be heterogeneously distributed on γδ T cell subsets with a bimodal distribution, possibly with functional significance [20].

In contrast, an observation made in our study was an exclusive infiltration of CD3^low^ Vγ9δ1 T cells in only RCC tumor tissues, and most of them were of the FAS^+^CD28^-^CD45RA^+^ T_EMRA_ cells. Moreover, this indicated that newly defined CD3^low^ Vγ9δ1 T cells could be predominantly in situ anti-RCC memory effectors compared to conventional peripheral circulating CD3^high^ Vγ9δ2 γδ T cells that have been generally utilized in ACT [7,8,9,10,11]. Notably, CD3 can be transiently and reversibly downregulated through internalization when activated by the TCR-CD3 cognate antigen [20]. CD3^low^ Vγ9δ1 T cells might have been activated by RCC-associated antigens still uncharacterized in the TME. Collectively, these data indicate that RCC-infiltrating CD3^low^ Vγ9Vδ1 T cells with an effector memory phenotype could be emerging as important candidates for adaptive immunotherapies against advanced RCC.

Upon target recognition, Vδ1 T cell-mediated killing occurs via perforin and granzymes using mechanisms similar to those of Vδ2 T cells [18]. Notably, LAMP1 and perforin were identified as the most representative cytotoxic molecules of RCC-infiltrating CD3^low^ Vγ9 δ1 T cells, whereas peripheral circulating CD3^high^ Vγ9 δ2 T cells only expressed high levels of granzyme A. Granzyme A is the first to become detectable during differentiation into memory cells, followed by granzyme B and later perforin [13], supporting our findings that most RCC-infiltrating CD3^low^ Vγ9 δ1 T cells and peripheral circulating CD3^high^ were T_EM_ and T_CM_, respectively. In fact, T cell exhaustion describes a state of late-stage differentiation usually associated with active prevention of functionality via ligation of negative signaling receptors on the cell surface, which can be recovered by manipulating extrinsic regulatory pathways, for example, by immune checkpoint blockade [16]. A state of reversible exhaustion could be viewed as a physiological mechanism facilitating the retention of antigen-specific T cells in the repertoire under chronic antigenic stimulation by tumor-associated antigens that cannot be cleared [7,8,9,11,15,16]. For example, although the expression of PD-1 is associated with T-cell exhaustion, PD-1 expression increases within hours of T-cell activation [3], suggesting that PD-1 expression is associated with effector function.

Despite recent data suggesting that Vδ1 T cells exhibit a radical new adaptive immunobiology [7,8,9,10,15], the use of Vδ1 T cells as adoptive therapy has been limited because, unfortunately, studies using Vδ1 PBLs may not reflect the immunobiology and exact roles of tumor-infiltrating Vδ1 cells [10,11]. Here, we present novel findings regarding the immunobiology of CD3^low^ Vγ9δ1 T cells isolated from patients with RCC, which are phenotypically and functionally distinct from Vγ9δ1 PBLs. Our study is the first to identify a distinct population of RCC-infiltrating CD3^low^ Vγ9δ1 T cells largely absent from the blood, suggesting that RCC selectively retains these subsets that adopt a terminally differentiated effector memory phenotype and are in a state of exhaustion as a result of chronic stimulation by RCC tumor cells [13,14]. It is unclear whether these distinct features stem directly from the nature of the clonotypes present and their antigenic targets or whether they reflect the influence of RCC TME that may also influence intrarenal retention. Further studies based on immune repertoire sequencing could allow us to probe in-depth immunological features and TCR repertoire of RCC-infiltrating CD3^low^ Vγ9δ1 T cells. For example, a large part of the Vδ2 compartment is reportedly made up of CD27^low^ CD45RA^high^ Vδ1+ T cells in liver tissue, which are predominantly clonally expanded [21]. This study provides strong evidence in support of the novel perioperative adoptive transfer of CD3^low^ Vγ9δ1 TIL for combating RCCs.

Nevertheless, an important unanswered question in RCC is the nature of the antigens that are targeted by CD3^low^ Vγ9δ1 T cells. In the context of cancer, Vδ1 γδ T cells recognize altered-self lipids presented by CD1d [10]. The abundance of lipid sulfatide and glycosphingolipids contributing to RCC progression [10,22,23,24,25] led us to hypothesize that these molecules present on CD1d in RCC may play an important role in activating CD3^low^ Vγ9δ1 T cells. A greater understanding of the relationship between different Vγ9Vδ1 TCR ligands, their mode of recognition, tissue expression, and regulation/dysregulation will undoubtedly provide novel therapeutic avenues and insights into RCC [21].

## 5. Conclusions

The results of our analysis of the RCC TILs and TCGA dataset support γδ T cell anti-tumor activity and confirm preferential infiltration of the RCC TME by the Vδ1 cell subset. Infiltrating Vγ9Vδ1 T cells likely undergo chronic stimulation within the RCC TME, producing a terminally differentiated effector CTL phenotype. Thus, Vγ9Vδ1 T cells may represent an excellent candidate for adoptive immunotherapy in high-risk patients with locally advanced RCC. Optimized techniques promoting clinical-grade expansion and purification of CD3^low^ Vγ9δ1 TILs with a high proliferative capacity, a more pronounced Th1 polarization, an increased cytotoxic capacity and secretion of cytokines, and combination with other immunotherapeutic strategies, such as immune checkpoint inhibitors, should also be considered, aiming to maximize their therapeutic potential.

## Figures and Tables

**Table 1:** Patient samples and clinical characteristics.

Pt.	Age	Sex	^a^ Surgery Type	Tumor Type	pT	^b^ Stage	^c^ Grade	Analysis
#1	45	Male	Radical	Clear cell	pT2a	2	Ⅲ	Figure 2
#2	82	Female	Radical	Clear cell	pT1b	1	Ⅲ	Figure 2
#3	63	Male	Radical	Clear cell	pT2a	2	Ⅱ	Figure 2
#4	43	Female	Radical	Clear cell	pT3a	3	Ⅲ	Figure 2
#5	72	Male	Radical	Clear cell	pT3a	3	Ⅲ	Figure 2
#6	89	Male	Radical	Clear cell	pT1b	1	Ⅲ	Figure 2
#7	66	Male	Radical	Clear cell	pT3a	3	Ⅲ	Figure 2
#8	61	Male	Radical	Clear cell	pT3a	3	Ⅲ	Figure 2/Figure 3
#9	44	Male	Radical	Clear cell	pT2a	2	Ⅳ	Figure 2/Figure 3
#10	62	Female	Partial	Clear cell	pT1a	1	Ⅱ	Figure 2
#11	72	Male	Partial	Clear cell	pT3a	3	Ⅳ	Figure 2
#12	75	Female	Radical	Clear cell	pT2a	2	Ⅱ	Figure 2/Figure 3
#13	40	Male	Radical	Clear cell	pT1b	1	Ⅱ	Figure 2/Figure 3/Figure 4
#14	56	Male	Radical	Clear cell	pT1b	1	Ⅲ	Figure 2/Figure 3/Figure 4
#15	29	Male	Radical	Chromophobe	pT2a	2	Ⅲ	Figure 3/Figure 4
#16	58	Male	Radical	Papillary	pT3a	3	Ⅲ	Figure 3/Figure 4
#17	61	Male	Partial	Clear cell	pT1a	1	Ⅱ	Figure 5
#18	58	Male	Radical	Clear cell	pT2a	2	Ⅲ	Figure 2/Figure 4/Figure 5
#19	60	Male	Partial	Clear cell	pT1a	1	Ⅱ	Figure 5
#20	62	Female	Radical	Clear cell	pT3b	3	Ⅳ	Figure 5

^a^ Surgery Type (Nephrectomy); ^b^ Stage (TNM); ^c^ Fuhrman grade.

**Table 2:** List of immune gene signatures for gene set enrichment analysis.

γδT	Th1	Th2	Th17	Treg	CTL	Exhausted T	M1 Macrophage		M2 Macrophage	
*TRGC2*	*CD3E*	*CD3E*	*CD3E*	*CD3E*	*CD3E*	*CD3E*	*IL12*	*CD40*	*ARG1*	*EGF*
*TRD*	*CD4*	*CD4*	*CD4*	*CD4*	*CD4*	*CD4*	*IL23*	*IDO1*	*ARG2*	*CTSA*
*CD3D*	*TBX21*	*GATA3*	*RORA*	*TGFB1*	*FASL*	*PDCD1*	*IL12*	*KYNU*	*IL10*	*CTSB*
*CD3E*	*IFNG*	*IL4*	*RORG*	*FOXP3*	*PRF1*	*LAG3*	*TNF*	*CCR7*	*CD32*	*CSTC*
*CD28*	*TNF*	*IL5*	*IL17A*	*IL2RA*	*GZMA*	*TIM3*	*IL6*	*CD45*	*CD163*	*CTSD*
*KLRK1*	*IL2*	*IL13*	*IL17F*	*IL10*	*GZMB*	*BTLA*	*CD86*	*CD68*	*CD23*	*TGFB1*
*KLRC1*	*IL12RB1*	*CCL13*	*IL21*	*CTLA4*	*GZMK*	*CTLA4*	*MHCII*	*CD115*	*CD200R1*	*TGFB2*
*KLRC2*	*IL12RB2*	*CXCL12*	*STAT3*	*MAF*	*IFNG*	*FAS*	*IL1B*	*HLA-DR*	*PD-L2*	*TGFB3*
*KLRC3*	*STAT1*	*TNF*	*BATF*				*MARCO*	*CD205*	*PDL1*	*MMP14*
*KLRC4*							*iNOS*	*CD14*	*MARCO*	*MMP19*
*KLRD1*							*IL12*		*CSF1R*	*MMP9*
*CD160*							*CD64*		*CD206*	*CLEC7A*
*NKG7*							*CD80*		*IL1RN*	*WNT7B*
*GZMB*							*CXCR10*		*IL1R2*	*FASL*
*FASLG*							*IL23*		*IL4R*	*TNFSF12*
*IL18RAP*							*CXCL9*		*CCL4*	*TNFSF8*
*CCL3*							*CXCL10*		*CCL13*	*CD276*
*CCL4*							*CXCL11*		*CCL20*	*VTCN1*
*CCL5*							*CD86*		*CCL17*	*MSR1*
*XCL1*							*IL1A*		*CCL18*	*FN1*
*XCL2*							*IL1B*		*CCL22*	*IRF4*
							*IL6*		*CCL24*	*CD45*
							*TNFa*		*LYVE1*	*CD68*
							*MHCII*		*VEGFA*	*CD115*
							*CCL5*		*VEGFB*	*HLA-DR*
							*IRF5*		*VEGFC*	*CD205*
							*IRF1*		*VEGFD*	*CD14*

## Data Availability

The data presented in this study are available on request from the corresponding author. The data are not publicly available due to privacy.
